# The significance of glycolysis in tumor progression and its relationship with the tumor microenvironment

**DOI:** 10.3389/fphar.2022.1091779

**Published:** 2022-12-14

**Authors:** Daoying Zhou, Zhen Duan, Zhenyu Li, Fangfang Ge, Ran Wei, Lingsuo Kong

**Affiliations:** ^1^ Department of Anesthesiology, The First Affiliated Hospital of USTC, Division of Life Sciences and Medicine, University of Science and Technology of China, Hefei, China; ^2^ Department of Provincial Clinical College, Wannan Medical College, Wuhu, China; ^3^ Function Examination Center, Anhui Chest Hospital, Hefei, China

**Keywords:** glycolysis, tumor microenvironment, immune cells, inflammatory factors, targeted therap

## Abstract

It is well known that tumor cells rely mainly on aerobic glycolysis for energy production even in the presence of oxygen, and glycolysis is a known modulator of tumorigenesis and tumor development. The tumor microenvironment (TME) is composed of tumor cells, various immune cells, cytokines, and extracellular matrix, among other factors, and is a complex niche supporting the survival and development of tumor cells and through which they interact and co-evolve with other tumor cells. In recent years, there has been a renewed interest in glycolysis and the TME. Many studies have found that glycolysis promotes tumor growth, metastasis, and chemoresistance, as well as inhibiting the apoptosis of tumor cells. In addition, lactic acid, a metabolite of glycolysis, can also accumulate in the TME, leading to reduced extracellular pH and immunosuppression, and affecting the TME. This review discusses the significance of glycolysis in tumor development, its association with the TME, and potential glycolysis-targeted therapies, to provide new ideas for the clinical treatment of tumors.

## 1 Introduction

Cancer is a massive global health challenge (Yang X et al., 2022), and is a major contributor to the universal disease burden. The global burden of disease is expected to continue to grow in the next 20 years. It is estimated that 23.6 million (95% UI, 22.2–24.9 million) new cases and 10 million (95% UI, 9.36–10.6 million) cancer-associated deaths occurred in 2019. According to the Global Burden of Disease cancer deaths, with a 26.3% (95% UI, 20.3%–32.3%) rise in novel cancer cases, and a 20.9% (95% UI, 14.2%–27.6%) elevation in cancer-related mortality since 2010, cancer ranked second to cardiovascular disease among global deaths in 2019 (Kocarnik et al., 2022). Owing to the persistence of the COVID-19 pandemic, and subsequent delays and disruptions in cancer screening, diagnosis, and therapy worldwide (Kocarnik et al., 2022), the number of avoidable cancer deaths has increased dramatically (Maringe et al., 2020). Hence, it is critical to elucidate the occurrence and development of tumors and clinical therapeutic targets to reduce the morbidity and mortality of cancer patients. Glycolysis is a process of oxidative glucose catabolism, whereby glucose is broken down to lactate with a small amount of ATP production under anaerobic conditions (Fuller and Kim, 2021). Recent studies have demonstrated the essential roles of glycolysis in numerous tumors (Wang L et al., 2022; Xu et al., 2022; Yang J et al., 2022). These include the regulation of tumor growth, invasion, chemoresistance, and the tumor microenvironment (TME) (Bi et al., 2021). Studies have revealed that the TME strongly modulates tumorigenesis, tumor development, metastasis, and therapeutic response (Xiao and Yu, 2021). The TME is a complex tumor-associated environment, specifically supporting the survival and development of the tumor cells. It includes tumor cells, various immune cells, secretory factors, and extracellular matrix (ECM). Tumor cells and the TME constantly interact with one another and co-evolve (Xiao and Yu, 2021). In this review, we mainly discuss the significance of glycolysis in tumorigenesis and tumor development, the relationship between glycolysis and various types of cells and cytokines within the TME, and glycolysis-targeted anti-tumor therapy.

## 2 Tumor cell metabolism and glycolysis

**FIGURE 1:**
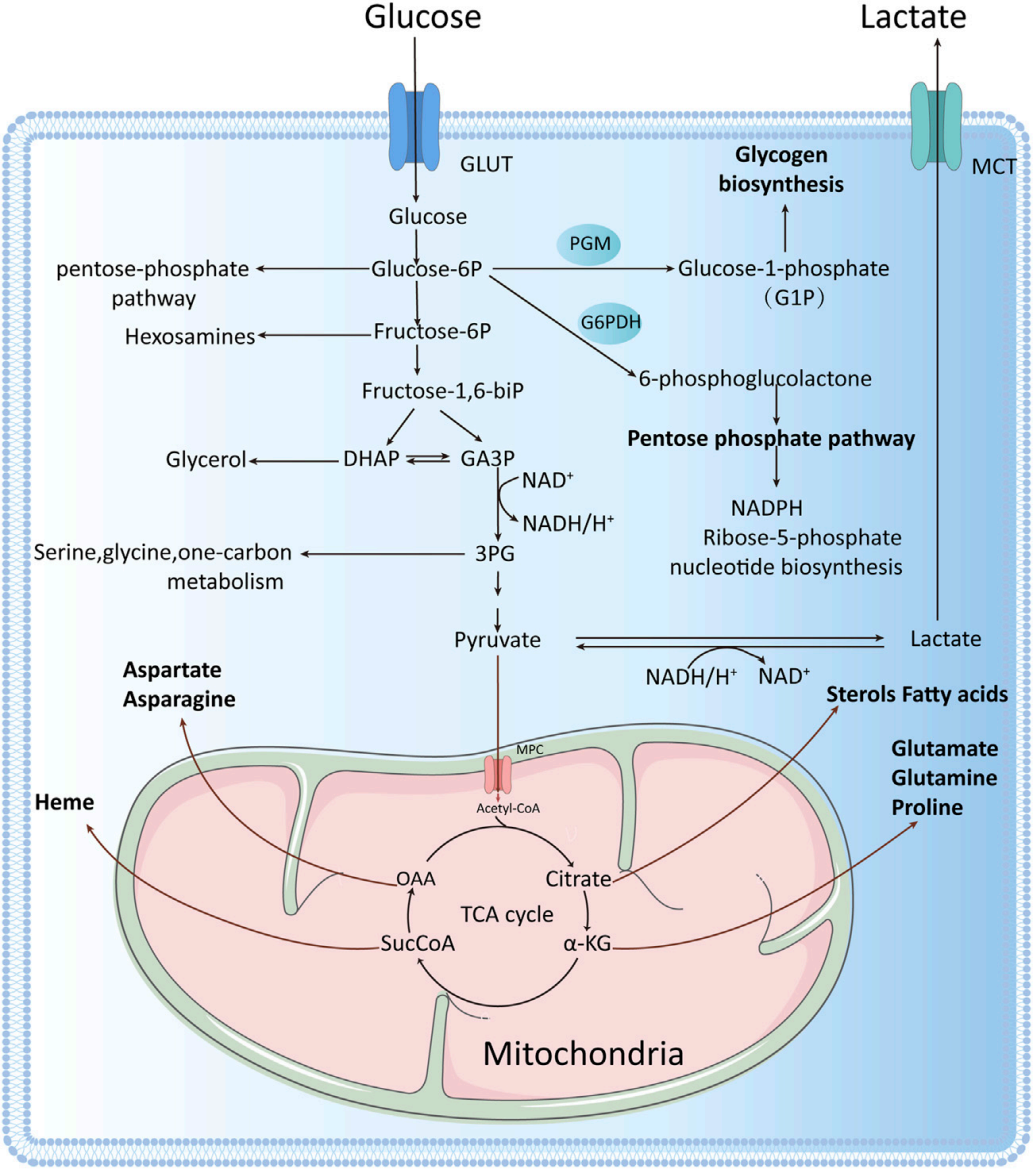
One glucose molecule is broken down intracellularly to two pyruvate molecules, which can enter the mitochondrion to participate in the tricarboxylic acid (TCA) cycle under aerobic conditions or to produce lactate under anaerobic conditions. Tumor cells rely mainly on aerobic glycolysis for energy production, even under oxygen-sufficient conditions, with one glucose molecule entering the glycolytic pathway to produce two net ATP molecules. Glycolysis involves the production of many intermediate metabolites apart from ATP. These intermediate metabolites can participate in other biosynthetic pathways, for example, glucose-6-phosphate can enter the pentose phosphate pathway, fructose 6-phosphate can enter the hexosamine biosynthesis pathway, dihydroxyacetone phosphate can be converted to glycerol 3-phosphate, and glycerol 3-phosphate can be converted to serine, cysteine, and glycine, among others.

Cellular energy is primarily derived from glucose conversion. Under conditions of sufficient oxygen, cells undergo aerobic oxidation, whereby glucose oxidizes to water and carbon dioxide. This is the main form of sugar oxidation, as well as the main process of cellular energy production. Under anaerobic conditions, however, glucose or glycogen breaks down to form lactate and energy. This process is known as anaerobic glycolysis (Figure 1).

Glycolysis has three main features: 1. It is the only way to produce ATP under anaerobic conditions or in cells with no mitochondria like erythrocytes and hyperthyroid cells. Under these circumstances, pyruvate is converted to lactate; 2. In presence of oxygen, glycolysis produces pyruvate, which enters into the tricarboxylic acid (TCA) cycle (also known as the citric acid or Krebs cycle) within the mitochondria to produce ATP; 3. Multiple glycolytic and TCA cycle metabolites can also become involved in anabolic networks to produce NADPH and intermediates required for glycogen, lipids, nucleotides and protein syntheses (Chandel, 2021). Thus, the primary goal of glycolysis is to produce intermediates that activate biosynthetic networks. Taken together, under oxygen availability, mitochondria provide a large amount of ATP to most cells. When oxygen is no longer available, glycolysis is activated to generate ATP for cell survival (Chandel, 2021).

Malignant tumor cells undergo excessive proliferation and detachment from neighboring cells to translocate to distant locations for metastasis. This requires ample energy and biosynthetic precursors that accelerate cell division, invasion, and migration. As an early tumor expands, it grows beyond the diffusion limit of local blood supply, which leads to hypoxia and upregulation of hypoxia-inducible transcription factor (HIF) expression (Infantino et al., 2021). Owing to the resulting reduced dependence on aerobic respiration, tumor cell metabolism generally switches to glycolysis by enhancing the production of glycolytic enzymes, glucose transporter proteins, and mitochondrial metabolic inhibitors (Hsu and Sabatini, 2008). By altering their energy metabolism, tumor cells gain a strong ability to survive in a hostile environment. This is known as “metabolic reprogramming”, and this mostly occurs due to enhanced glycolysis (Hsu and Sabatini, 2008).

Under oxygenated conditions, normally differentiated cells maximize ATP synthesis *via* mitochondria-mediated glucose oxidative phosphorylation. In contrast, tumor cells consume excess glucose and synthesize massive quantities of lactate, thereby relying on glycolysis for energy production even in a well-oxygenated environment (Zahra et al., 2020). This process is aerobic glycolysis or the Warburg effect (Wong et al., 2013). Although glycolysis is considerably less efficient in producing ATP per molecule of glucose, it produces ATP at a faster pace than oxidative phosphorylation. Thus, the unlimited tumor cell proliferation is amply supplied with rapidly producing energy (Zhang et al., 2020a; Domiński et al., 2020). Therefore, glycolysis is a critical element in tumor progression.

## 3 The significance and related mechanism of glycolysis in tumorigenesis and tumor development

Tumor cells exhibit a specific pattern of glucose metabolism, namely aerobic glycolysis, which is critical for tumorigenesis and tumor development, and it affects tumor growth, invasion, chemoresistance, and the TME (Bi et al., 2021). In pancreatic ductal adenocarcinoma, glycolysis was shown to promote tumor invasion and migration *via* large amounts of substrate production, and glycolytic enzymes and actin association, which ultimately support vigorous tumor cell growth (Yang et al., 2020). In addition, essential enzymes and glycolysis intermediates may modulate pancreatic ductal adenocarcinoma metastasis *via* participation in colonization-related networks, angiogenesis, epigenetic mechanisms, or activation of the epithelial-mesenchymal transition (Tsutsumi et al., 2004; Azoitei et al., 2016; McDonald et al., 2017; Liu et al., 2021). Non-coincidentally, Deng et al. (2019) also reported that aerobic glycolysis is essential for angiogenesis in colorectal cancer (CRC) cells, and angiogenesis is a critical modulator of tumor progression and metastasis. In CRC cells, glucose-derived aerobic glycolysis-generated lactic acid accumulates in the TME, wherein it stimulates the formation of vascular endothelial cells, which, in turn, promote tumor progression and metastasis. CRC cells also rely on aerobic glycolysis-derived ATP for rapid growth and chemoresistance (Wang et al., 2020). Emerging reports suggested that hypoxic tumors with enhanced glycolysis are more prone to metastasis than normoxic tumors. Therefore, augmented glycolysis is a major contributor to metastasis, and is a poor prognostic phenotype in cancers such as prostate cancer (Ghanavat et al., 2021).

Impaired energy metabolism and immune evasion are two major characteristics of cancer. Cancer cells employ the glycolytic network for energy production and reprogram the TME *via* the abundant energy supply (Chang et al., 2015; Ganapathy-Kanniappan, 2017; Vaupel et al., 2019). Multiple reports have suggested that tumor metabolism and tumor-based immune evasion are dependent on one another (Cascone et al., 2018). On the one hand, Jiang et al. (2019) revealed a strong direct association between tumor glycolysis and tumor immunity in various cancers, with glycolytic activity being more predictive of immune signaling in different cancers compared to tumor mutational burden and tumor aneuploidy. In addition, glycolysis also increased the programmed cell death ligand 1 (PD-L1) content in tumor cells, thereby enhancing the anti-PD-1/PD-L1 immunotherapeutic response. Tumors with enhanced glycolysis exhibit better immunotherapeutic response and good survival in an immunotherapeutic setting. Therefore, tumor glycolytic activity is a potential prognostic indicator for immunotherapeutic response in a variety of cancers (Jiang et al., 2019). On the other hand, Li et al. (2020) reported a strong association between enhanced glycolytic activity and pro-tumor immunity/inflammation in breast cancer. In the high-glycolysis group, there was a marked elevation in the tumor immunity/inflammation-associated gene expression and immune/inflammatory networks, particularly the IL-17 axis. Moreover, several immune/inflammatory cells such as Th2 cells and macrophages were enriched, whereas the invasion of killer immune cells such as NKT cells was diminished, and the immune checkpoint genes PD-L1, CTLA4, FOXP3, and IDO1 were elevated. This evidence suggests that tumor glycolysis can accelerate tumor immunity/inflammation *via* the IL-17 axis to enhance the immune/inflammatory functions of tumor cells (Li et al., 2020).

Furthermore, glycolytic activity is also closely related to apoptosis, a strong direct indicator of immune function. This indicates that apoptosis may serve as a bridge between glycolytic and immune activities in tumors (Jiang et al., 2019). It was revealed that increased aerobic glycolysis in nasopharyngeal carcinoma cells *via* the IRF2/CENP-N/AKT axis promoted malignant biological behavior while inhibiting apoptosis (Qi et al., 2021). In hepatocellular carcinoma (HCC), aerobic glycolysis was also found to inhibit Silibinin-triggered apoptosis in human HCC HepG2 and Hep3B cells (Yang et al., 2021). In addition to the glycolysis-mediated suppression of tumor cell apoptosis, its metabolite lactate accumulates in the TME, leading to acidification of the extracellular pH and immunosuppression, which, in turn, affect the TME (de la Cruz-López et al., 2019).

## 4 The TME

**FIGURE 2:**
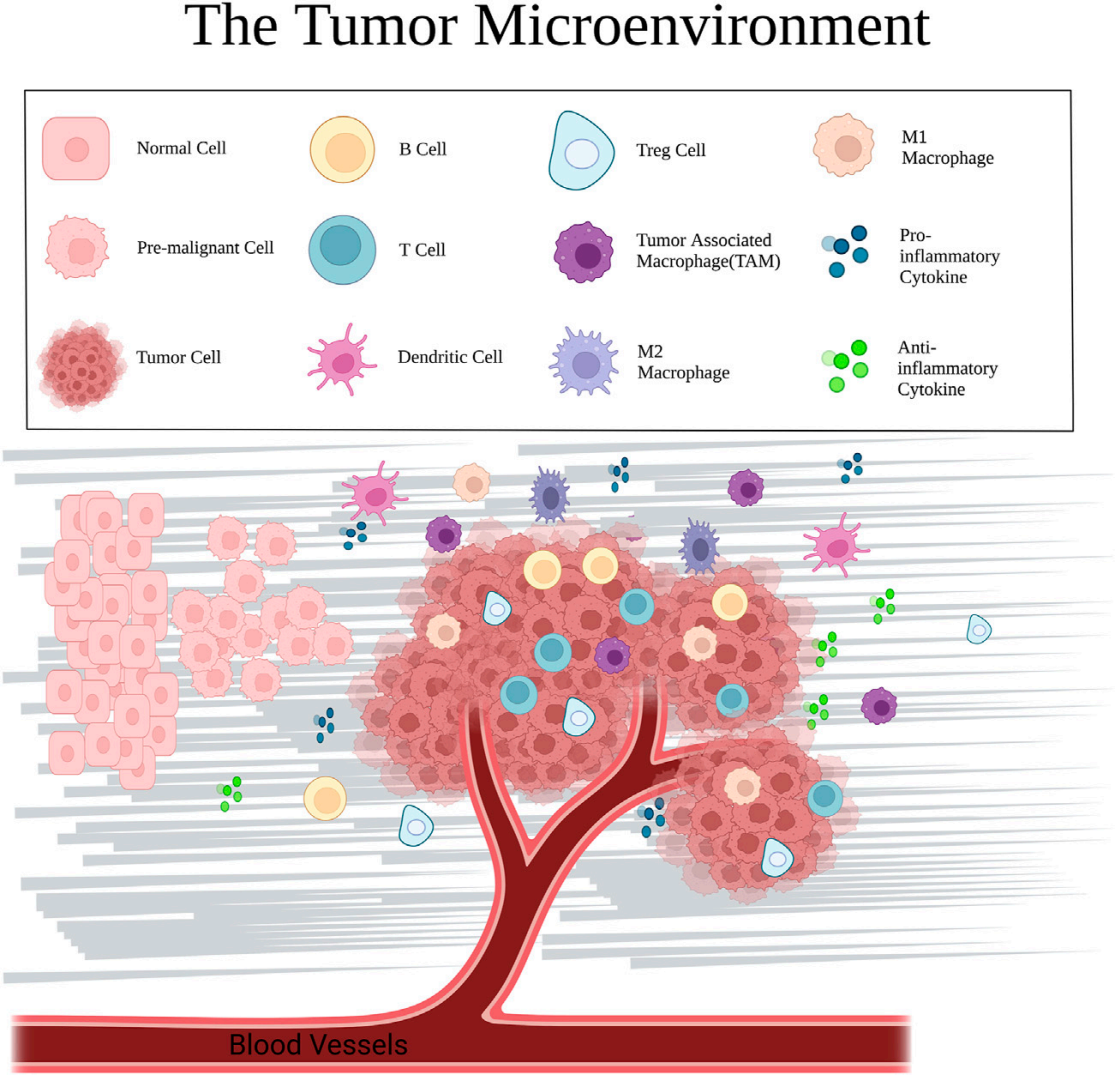
Major cellular components and mediators in the TME, including normal cells, cancer cells, immune cells (T cells, B cells, dendritic cells, TAMs, and macrophages), cytokines, and extracellular matrix.

Cell proliferation and apoptosis in normal tissues remain in a state of equilibrium. Disruption of this balance can result in the development of various benign and malignant neoplastic diseases. The TME is a cellular niche comprising a dynamic heterogeneous collection of tumor or cancer stem cells, as well as being involved in the constant modulation of infiltrating and resident host cells, secretory factors, and ECM (Xiao and Yu, 2021). This niche not only includes the structure, function and metabolism of the tumor-host tissue, but also the internal environment (nucleus and cytoplasm) of the tumor cells themselves (Anderson and Simon, 2020; Cao et al., 2022). The cellular components of the TME include the tumor cells themselves, as well as adipocytes, fibroblasts, tumor vascular system, lymphocytes, dendritic cells, and tumor-related fibroblasts (Figure 2), each of which has a unique immune capacity that will determine the survivability and influence of tumor cells on neighboring cells (Arneth, 2019). The non-cellular components include chemokines and cytokines (for example, IL-1β, IL-33, IL-6, TNF-α, and IL-17). The cellular and non-cellular components together form a complex TME that synergistically supports tumor growth (Arneth, 2019). Parenchymal and mesenchymal cells are morphologically, phenotypically, and functionally distinct among patients, between primary and metastatic tumors, and even within individual tumors at multiple levels (Naxerova et al., 2014; Naxerova and Jain, 2015). Tumor cells cross-talk with neighboring cells using the circulatory and lymphatic systems to regulate tumor development. During tumor cell proliferation, tumor cells recruit surrounding non-tumor cells (Balamurugan, 2016) to foster a specific TME that facilitates local tumor development and metastasis to distant organs. These tumor cells associate with host cells to generate an aberrant organoid structure (Ge and Ding, 2020; Uneda et al., 2021). The available literature establishes TME as an essential niche for tumor development, heterogeneity among and within tumors, and systemic therapy resistance (Jin and Jin, 2020; Wang and Ilyas, 2021). It is well established that glycolysis modulates the TME, and, in recent years, numerous studies have reported links among glycolysis, immune cells, and inflammatory factors within the TME.

## 5 Relationships between glycolysis and immune cells in the TME

Glycolysis is inseparable from the immune cells present in the TME. Macrophages are specialized phagocytic cells belonging to the innate immune system, and they serve an essential function in the TME, along with their ability to fight inflammation. Macrophages are typically classified as M1 and M2 types, with M1-type macrophages promoting inflammatory responses, and M2-type macrophages promoting tumor development (Jablonski et al., 2015). The M2-type macrophages differentiate to form tumor-associated macrophages (TAM), which are critical regulatory cells for tumor immunity and immunotherapy (DeNardo and Ruffell, 2019). Glycolysis produces lactate, a pro-tumor metabolite, and lactate promotes M2-like gene expression in M1 macrophages, and enhances PD-L1 expression in M1 macrophages, which leads to an M1 phenotype that is both pro-inflammatory and potentially oncogenic (Morrissey et al., 2021). In HCC, cancer cell-based fibronectin 1 (FN-1) activates glycolysis within macrophages by triggering TLR4, which results in the significant upregulation of pyruvate kinase M2 (PKM2), which, in turn, enhances the syntheses of IL-1β, IL-12p70, TNF-α, HLA-DR, and PD-L1. In addition to being a glycolytic rate-limiting enzyme, PKM2 enters the nucleus to maintain HIF-1α stability, while inducing macrophage polarization, which diminishes the anti-tumor activity of macrophages (Lu et al., 2022). Similarly, lactate accumulation in breast cancer contributes to the M2-type macrophage polarization, which promotes tumor development both *in vivo* and *in vitro*. Additionally, this is correlated with worse outcomes in breast cancer patients (Jiang et al., 2022). It was revealed that, in pancreatic cancer (PC), the TAM paracrine factor CCL18 promotes malignant tumor progression and induces glycolysis by upregulating VCAM-1. In contrast, VCAM-1-triggered lactate formation in PC cells enhances aerobic glycolysis while activating macrophages to form a TAM-like phenotype, creating a positive feedback loop (Ye et al., 2018). The first rate-limiting enzyme in glycolysis is hexokinase. Hexokinase 3 (HK3) expression was shown to be strongly correlated with macrophage and dendritic cell infiltration. Moreover, it can promote the progression of renal clear cell carcinoma (Xu et al., 2021). Fructose-2,6-bisphosphatase 3 (PFKFB3) is a glycolytic activator, and PFKFB3 upregulation in peritumor-associated monocytes/macrophages in HCC promotes tumor progression *via* attenuation of the cytotoxic T lymphocyte response in tumor tissue using the PFKFB3/NF-κB/PD-L1 axis (Chen D.P et al., 2019).

Lymphocytes are another critical element for the TME. Moreover, the antitumor activity of lymphocytes is influenced by multiple factors. Cancer cell-mediated glycolysis is known to impair T cell activation and antitumor responses. For example, lactic acid accumulation impairs the activation and migration of T cells and promotes the immune escape of tumor cells (Beckermann et al., 2017). Among them, regulatory T cells (Tregs) strongly modulate homeostasis of the immune system, body immune tolerance, enhanced angiogenesis, tumor growth, and proliferation, as well as tumor transformation into metastatic disease (Paluskievicz et al., 2019). PD-1 expression of Treg cells is markedly increased in highly glycolytic tumors, relative to effector T cells (Kumagai et al., 2022). Tumor glycolytic activity is directly associated with PD-L1 levels and the immune response. Hence, tumor glycolytic activity can serve as a marker for tumor immunotherapeutic response prediction (Jiang et al., 2019).

NK cells exhibit antitumor cytotoxicity. Moreover, glycolysis-induced lactate production and the acid environment resulting from the excess lactate markedly reduce NK cell cytotoxicity, causing them to lose their antitumor function. In addition, the nuclear factor of activated T cells (NFAT) axis-related genes are suppressed, which leads to a decrease in NFAT-regulated IFN-γ production, an essential factor for the hypoxia-induced accelerated glycolysis in cancer cells (Brand et al., 2016; Hasan et al., 2022). One study revealed that glycolysis-related genes are highly enriched in advanced cancer patients, particularly, when tumors undergo hypoxia. Moreover, pre-activated NK cell regulation *via* the ERK/STAT3 pathway enhances NK cell proliferation and cytotoxicity (Lim et al., 2021). Knockdown of CIS, an intracellular protein, in induced pluripotent stem cell-derived NK cells (iPSC-NK cells) improves metabolic adaptations, as evidenced by enhanced basal glycolytic and glycolytic capacities, which contributes to marked increases in NK cytotoxicity and antitumor activity (Zhu et al., 2020).

Dendritic cells (DCs) are antigen-presenting cells, and are responsible for the capture of pathogen- or tumor-associated antigens for T cell presentation to induce an immune response. However, the DC-based immune activity in the TME is usually suppressed. Moreover, lactate activates G protein-coupled receptors (GPR81) on murine DCs (which inhibit MHC II presentation on the DC surface), as well as the lactate-induced acidic TME (which inhibits antigen uptake by DCs and stabilizes antigen-MHC-I complexes) to promote tumor escape (Peng et al., 2021). Furthermore, HIF-1α and c-Myc convert more pyruvate to lactate by interacting with the lactate dehydrogenase (LDH-A) promoter in the hypoxic environment within tumor cells, and the resulting lactate accumulation within the TME further suppresses DC activation and antigen expression (Burgdorf et al., 2020).

## 6 Relationship between glycolysis and inflammatory factors in the TME

Inflammatory cells are also present in the TME, where they modulate tumor growth by the secretion of active molecules (such as IL-1β, IL-33, IL-6, TNF-α, and IL-17) into the TME. Moreover, chronic inflammation leads to the reprogramming of the tumor cell glucose metabolism to promote tumorigenesis (Vaughan et al., 2013). The IL-1 family encompasses essential inflammatory cytokines such as IL-1α, IL-1β, and IL-33, which modulate immune and inflammatory responses and regulate glycolysis by activating the glycolytic enzymes HK, glucokinase, PFK, and LDHA (Tan et al., 2018). IL-1β has been intensively studied, and IL-1β production by M2 macrophages promotes glycerol-3 -phosphate dehydrogenase (GPD2) phosphorylation, which, in turn, accelerates the glycolytic rate to promote glioma cell proliferation (Lu et al., 2020). IL-1β also promotes glycolysis in lung adenocarcinoma cells *via* the p38 axis, which further enhances lung adenocarcinoma cell migration and invasion (Tan et al., 2021). IL-33, on the other hand, accelerates Th2-associated cytokine synthesis and upregulates glucose transporter protein 1 (GLUT1) through the IL-33/ST2 pathway to enhance glucose uptake and glycolysis in tumor cells, while promoting growth and metastasis of non-small cell lung cancer (Wang et al., 2016).

Expression of the pro-inflammatory cytokine IL-6 is significantly elevated in numerous malignancies and is correlated with poor survival outcomes in several cancer types. IL-6 is a major modulator of the STAT3 signaling pathway, and it activates Glut5 by triggering the STAT3 axis to regulate fructose metabolism and tumorigenesis (Huang et al., 2022). Chen X et al. (2019) reported that IL-6 stimulates PC cell proliferation, survival, and glycolysis *via* the GP130/STAT3 axis. Zhang et al. (2021) also revealed that the immunoglobulin superfamily containing leucine-rich repeat (ISLR) gene deficiency markedly reduced JAK2 and STAT3 phosphorylation, thereby inhibiting the IL - 6/JAK/STAT3 axis, which, in turn, accelerates apoptosis, and suppresses non-small cell lung cancer cell proliferation, migration, invasion, and glycolysis. In addition, IL-6 was also shown to enhance glycolysis by upregulating the expression of the glycolytic enzyme PFKFB3 to promote colorectal carcinogenesis and progression (Han et al., 2016). Zhang et al. (2018) observed that the IL-6-induced enhancement of phosphoglycerate kinase 1 (PGK1) threonine (T) 243 phosphorylation was mediated by the 3-phosphatidylinositol-dependent protein kinase 1 (PDPK1) in tumor cells. This phosphorylation enhances PGK1-catalyzed glycolytic reactions by modulating their substrate affinities and has been correlated with human glioblastoma multiforme (GBM) malignancy and prognosis.

TNF-α and IL-17 are produced ubiquitously in acute and chronic inflammation, and both are associated with CRC. TNF-α and IL-17 synergistically enhance glycolysis and lactate production in CRC HT-29 cells *via* activation of the NF-κB axis in tumor cells, and they promote GLUT1 and hexokinase 2 (HK2), as well as expression of the common target genes HIF-1α and c-myc which, in turn, promote tumorigenesis (Straus, 2013). Emerging reports have suggested that the treatment of human colon cancer adenocarcinoma with the two pro-inflammatory cytokines, TNF-α and IL-17, also altered LDH activity, leading to an LDH shift to the A isoform, LDH-A, a major modulator of aerobic glycolysis. This effectively reduces pyruvate and enhances lactate levels in tumor tissue, thereby increasing cancer cell migration. Meanwhile, these two cytokines also induce the epithelial-mesenchymal transition characteristics of human colon cancer adenocarcinoma cells, including decreased e-calcineurin levels and increased metalloproteinase secretion (Baumann et al., 2009; Manerba et al., 2017). The stimulatory role of glycolysis in tumorigenesis and tumor progression has brought the glycolytic pathway into the public eye as a potential therapeutic target of anti-tumor therapy.

## 7 Glycolysis-targeted cancer therapy

**FIGURE 3:**
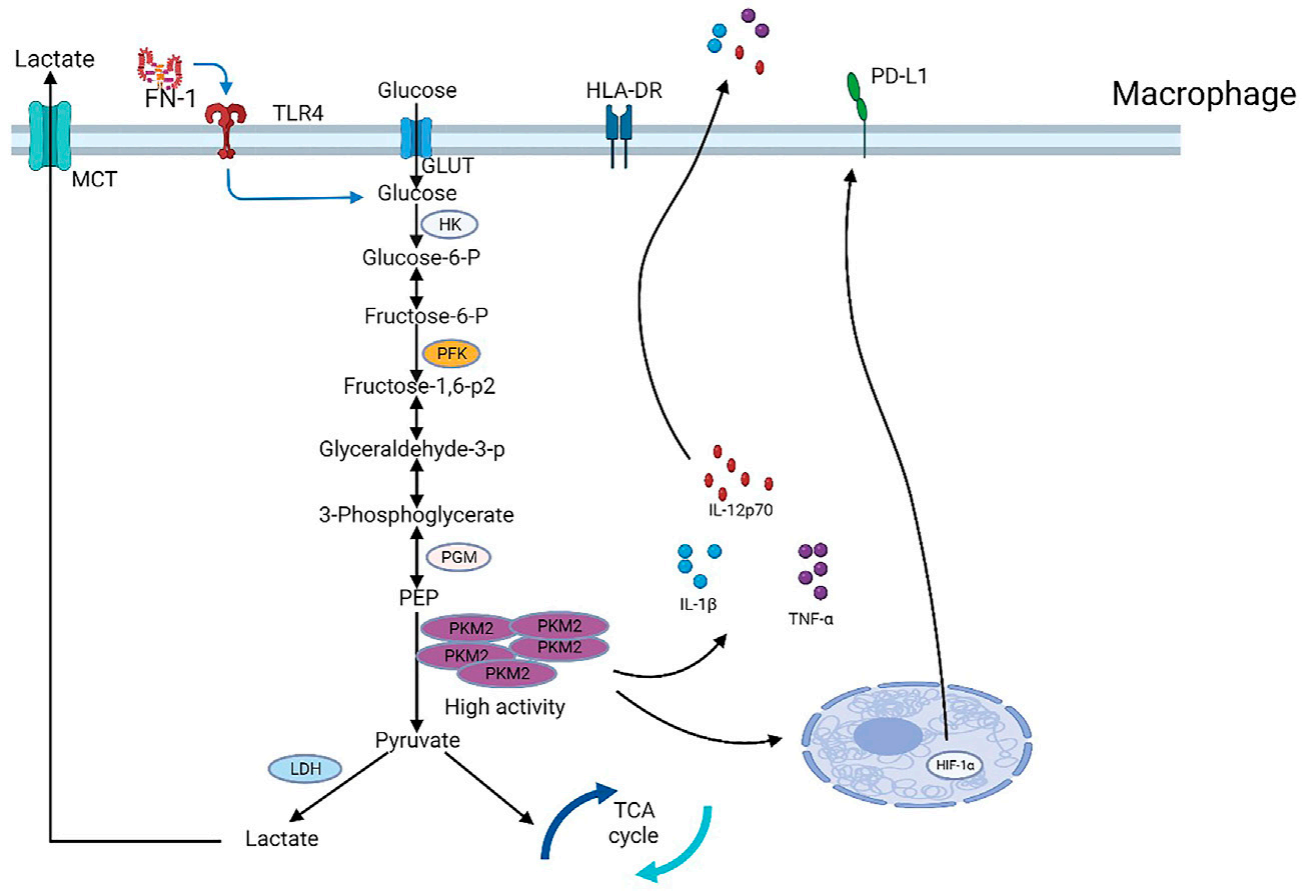
Major rate-limiting enzymes, metabolic enzymes, and transporters in the glycolytic pathway. PKM2, a key glycolytic enzyme regulated by fibronectin 1 (FN-1) secreted by hepatocellular carcinoma cells, controls PD-L1 expression in macrophages in a HIF-1α-dependent manner.

Aerobic glycolysis is a critical metabolic property of tumor cells, and it provides rapid energy, essential precursors for a variety of other metabolic pathways, and raw materials for the synthesis of multiple biomolecules (Pavlova and Thompson, 2016). More significantly, enhanced glycolysis within tumor cells results in the synthesis of excess lactate which, along with the reduced glucose metabolic environment, sustains the immunosuppressive TME (Ippolito et al., 2019). The aerobic glycolysis phenomenon is further accompanied by an upregulation of glycolysis-related rate-limiting enzymes, transporters, and other metabolic enzyme levels, namely, GLUT1, HK2, phosphofructokinase 1 (PFK1), PKM2, LDH-A, and monocarboxylate transporter protein 1 (MCT1) (Tanner et al., 2018) (Figure 3).

GLUT1 is a glucose transporter protein that plays a role in transporting glucose or fructose in cells. Xiao et al. (2018) showed that GLUT1 knockdown inhibits prostate cancer growth by suppressing tumor-cell glycolysis and proliferation while promoting cell cycle arrest. Similarly, other researchers reported that GLUT1 deficiency also inhibited CRC cell proliferation, migration, and glycolysis (Zhang et al., 2020b), while reversing the Warburg effect in gastric cancer cells, while enhancing cellular apoptosis (Dai et al., 2021). Dihydroartemisinin and rhodopsin are also reported to exert anticancer effects by inhibiting GLUT1 (Gao et al., 2020; Wang et al., 2021). Furthermore, HK is the first rate-limiting enzyme in the glycolytic reaction. It converts glucose to glucose-6-phosphate (G-6-P). HK2 is an isoform of HK, and in human glioblastoma cells, elevated glucose levels induce the mitochondrial separation of HK2, which then phosphorylates IκBα, and causes its destruction, thereby transcriptionally enhancing PD-L1, which promotes tumor cell immune evasion and brain tumor development (Guo et al., 2022). It is reported that HK2 deficiency significantly inhibits glycolysis and tumor cell growth in HCC (Wu et al., 2019), while HK2 activity suppression promotes cell death in glioblastoma (Uludağ et al., 2022). Current studies have demonstrated that 2-deoxy-D-glucose (2-DG) and 3-bromopyruvate (3-BrPA) can act as inhibitors of HK2 and inhibit the glycolytic process of tumor cells, thereby exerting an anti-tumor effect (Fan et al., 2019; Pajak et al., 2019).

PFK1 is the second rate-limiting enzyme in the glycolytic process. It was found that siRNA-based PFK1 deficiency enhanced apoptosis and inhibited rectal cancer cell migration and proliferation (Tian et al., 2020). Moreover, the stable downregulation of PFK1 expression suppresses human nasopharyngeal carcinoma CNE2 cell development, while inducing apoptosis, and reducing cell invasion and metastasis (Li S et al., 2021). So, PFK1 is a promising new target for nasopharyngeal and rectal cancer therapy, and it has great potential that can be extended to other malignancies. The activity of PFK1 is regulated by PFKFB3. PFKFB3 catalyzes fructose 6-phosphate to produce fructose 2,6-diphosphate, which is a metabotropic activator of PFK1 and can significantly increase the catalytic activity of PFK1. There are a number of PFKFB3 inhibitors developed so far, including KAN0438757, 3PO, and PFK15 (Hu et al., 2020; De Oliveira et al., 2021; Yan et al., 2021). Moreover, PKM2 is an enzyme that modulates the final rate-limiting glycolytic step, and it is a critical modulator of tumor metabolism. PKM2 is overexpressed in multiple cancers, and it promotes tumor cell proliferation and metastasis (Zhu et al., 2021). PKM2 methylation reversibly shifts the metabolic balance in breast cancer cells from oxidative phosphorylation to aerobic glycolysis, whereas inhibition of PKM2 methylation disrupts this intricate balance in cancer cells and suppresses breast cancer cell proliferation, migration, and metastasis (Liu et al., 2017). Recent studies have reported that shikonin and its analogs, flavonoid derivatives, 2,3-dithiocarbamate substituted naphthoquinones, benserazide, and Compounds 3 k and 10i, can act as inhibitors of PKM2 and inhibit aerobic glycolysis of tumor cells to exert anti-tumor effects (Zahra et al., 2020; Zhu et al., 2021).

LDH-A is a cytoplasmic enzyme that is primarily involved in anaerobic and aerobic glycolytic processes, and elevated LDH-A levels are strongly correlated with worse prognosis among tumor patients (Valvona et al., 2016). It was reported that reduced LDH-A expression suppresses glycolysis, tumor growth, and lung metastasis in thyroid cancer *in vitro* and *in vivo* (Huo et al., 2021). LDH-A knockdown in triple-negative breast (TNBC) cell lines impairs the aerobic glycolytic process, thereby significantly inhibiting the proliferation, migration, and invasion of TNBC cells (Wang W et al., 2022). Currently, gossypol (AT-101) and its derivatives FX-11, galloflavin, and N-hydroxy indole-based compounds have been shown to selectively and preferentially inhibit LDHA and suppress tumor progression (Ippolito et al., 2019). Moreover, MCTs modulate glycolytic metabolism and tumor cell survival using a proton-linked transmembrane lactate transport, and it was revealed that the pharmacological inhibition of MCT1 effectively inhibits glioma angiogenesis, which leads to a reduction in glioma cell growth, proliferation, and migration (Miranda-Gonçalves et al., 2013). Similarly, the siRNA-mediated MCT1 knockdown suppresses kidney cancer cell proliferation and migration, thereby suppressing tumor progression (Li M et al., 2021). To date, α-cyano-4-hydroxycinnamate (CHC), phloretin, quercetin, AZD3965, and AR-C155858 have been found to have some anticancer potential as inhibitors of MCT1 (Puri and Juvale, 2020). Therefore, targeting GLUT1, HK2, PFK1, PKM2, LDH-A, or MCT1 in various tumors may be an effective approach for treating tumors, and all these possibilities have the potential to be effective therapeutic targets against tumors.

In addition, there are numerous other therapeutic targets for tumors. For example, mTOR is a major direct regulator of the Warburg effect, while HIF1α, a downstream mTOR target, is highly expressed and synergizes with c-Myc-hnRNP splicing modulators to enhance PKM2 expression, which, in turn, promotes tumor progression. Thus, the entire mTOR/HIF1α/Myc -hnRNPs/PKM2 axis components can serve as potential glycolytic therapeutic targets for tumors (Sun et al., 2011). Others also revealed that PD-L1 is highly expressed in Tregs and that PD-L1 inhibition in Tregs or Tregs may be an effective measure against cancer (Kim et al., 2019). CTLA-4 is constitutively expressed in tumor-infiltrating Tregs, while it is only expressed in low amounts of the surfaces of circulating Tregs and lymphoid organs. Therefore, it can also be selectively used in tumor tissues with anti-CTLA-4 antibodies (Paluskievicz et al., 2019). Drugs for these therapeutic targets are being researched and developed, and a growing number of researchers are dedicating themselves to them.

## 8 Conclusion

In summary, TME-based glycolysis is closely related to a variety of immune cells, namely, macrophages, T lymphocytes, NK cells, DCs, and inflammatory cytokines such as IL-1β, IL-33, IL-6, IL-17, and TNF-α. Together, these promote tumor development, invasion, metastasis, chemoresistance, and regulate tumor immune function. Recent studies have revealed that tumor glycolytic activity can also serve as a biomarker for tumor immunotherapeutic response prediction. Given that aerobic glycolysis is critical for tumor progression, the targeting of aerobic glycolysis has become a current research hotspot for the development of new anti-cancer treatments. Tumor cells mainly rely on aerobic glycolysis for the specific reprogramming of glucose metabolism, and many metabolic enzymes are involved in the glycolytic process. Targeting these metabolic enzymes to inhibit tumor progression requires the design of drugs that specifically target metabolic enzymes in the tumor, thus inhibiting tumor progression and minimizing the side effects on normal tissues. Targeted glycolytic therapy may enhance the clinical treatment of tumor patients through its combination with other therapeutic approaches, such as immune- and chemotherapies. It is critical to gain a deeper knowledge of the mechanism by which glucose metabolism is altered in tumors and how it regulates cancer cells. Hence, further investigation into the metabolic enzymes associated with glycolysis as therapeutic targets in tumors is warranted. This requires the design and development of highly specific and selective inhibitors for these enzymes and the development of treatment protocols that both improve efficacy and reduce toxicity when combined with other chemotherapeutic agents. This will help create a positive atmosphere for the fight against cancer and will bring new hope to patients with cancer.
